# Hyperacute Therapies for Childhood Stroke: A Case Report and Review of the Literature

**DOI:** 10.1155/2010/497326

**Published:** 2010-08-19

**Authors:** Jose Irazuzta, Kevin J. Sullivan

**Affiliations:** ^1^Division of Pediatric Critical Care Medicine, University of Florida Health Science Center at Jacksonville and The Wolfson Children's Hospital, 800 Prudential Drive, Jacksonville, FL 32207, USA; ^2^Department of Anesthesia, Mayo Clinic Rochester, Rochester, MN 55905, USA

## Abstract

*Objective*. The optimal management of pediatric patients with arterial ischemic stroke (AIS) is not known. Despite this, goal-oriented, time-sensitive therapies geared to rapid reestablishment of arterial blood flow are occasionally applied with beneficial effects. The inconsistent approach to AIS is in part due to a lack of knowledge and preparedness. *Methods*. Case report of a 12-year-old male with right middle cerebral artery (MCA) occlusion resulting in dense left hemiplegia and mutism and review of the literature. *Intervention(s)*. Mechanical thrombectomy, intra-arterial administration of rt-PA, vasodilators, and platelet inhibitors, and systemic anticoagulation and subsequent critical care support. *Results*. Restoration of right MCA blood flow and complete resolution of neurologic deficits. *Conclusion*. We report the gratifying outcome of treatment of a case of AIS in a pediatric patient treated with hyperacute therapies geared to arterial recanalization and subsequent neurologic critical care and review the pertinent literature. Guidelines for the emergency room management of pediatric AIS from prospective, randomized trials are needed.

## 1. Introduction

Arterial ischemic stroke (AIS) occurs more commonly in adults than children. In children the clinical presentation is often interpreted as other neurologic conditions or intoxications resulting in a delay in diagnosis due to the lack of awareness of pediatrician or that the symptoms do not systematically evoke a stroke. Adult AIS occurs in the setting of the principal risk factors of hypertension and atherosclerosis, while pediatric risk factors are heterogeneous and include focal intracranial arteriopathies, congenital cardiovascular diseases, and hemoglobinopathies [1, 2]. Recent advances in the management of AIS have emphasized the importance of timely diagnosis and restoration of arterial flow in the affected vascular territories. Adult victims have access to time-sensitive or hyperacute therapies due to an awareness of the severity of the disease, organization of delivery of medical care from the emergency room that includes designation of stroke centers, and protocol-driven preparedness for such emergencies [3–6]. Unfortunately, this is not the case for pediatric victims, because it is not known whether thrombolytic therapies are appropriate. 

We describe the case of a 12-year-old male with AIS who presented to medical attention with a potentially devastating neurological injury. A resolute strategy utilizing hyperacute therapies aimed at restoring blood flow followed by neurointensive care support was provided. The patient had a very good outcome that may be related to this management but a complete causation cannot be ascribed to it as 1 out of 3 patients with lenticulostriate stroke may have a spontaneous favorable evolution. Herein we review the literature to date describing the challenges in analyzing and preparing for the application of time-sensitive therapies in the treatment of AIS.

## 2. Case Report

The institutional IRB was consulted and waved the need for approval of the publication of this case. The patient is a 12-year-old, right-handed, 52 kg African-American male without any significant medical history other than nocturnal enuresis. His father woke him up at 04:00 on the morning of admission as was customary in their home to prevent bedwetting. While initially he responded appropriately, the child collapsed to the ground after standing and became unresponsive. He was transported to an outlying emergency department where he was noted to have aphasia and left dense hemiplegia. Computed tomography demonstrated signal attenuation and edema in the vascular distribution of the right middle cerebral artery. Right lateral ventricle compression and midline shift were noted. 

**Figure 1:**
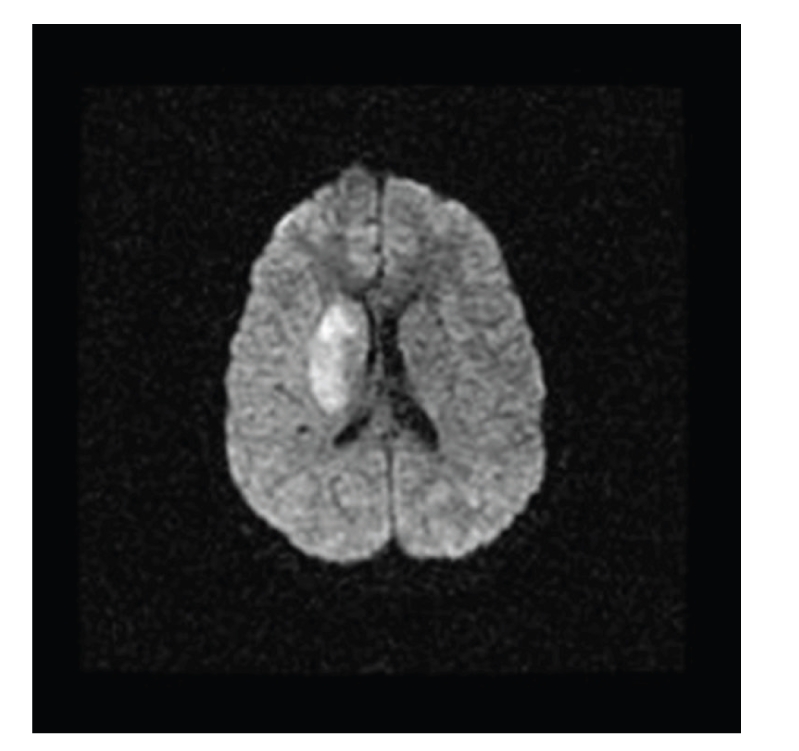
Diffusion weighted imaging (DWI) showing area of restricted diffusion.

The patient was transferred to our institution by helicopter and was briefly examined in the emergency room prior to transfer to the magnetic resonance (MR) imaging suite. He was awake, with stable airway, anxious and frustrated by inability to speak, but was cooperative and able to follow some commands. Heart rate was 50–60 beats per minute and blood pressure was 130–140/80–90 mm Hg. He demonstrated lack of language expression, flaccid paralysis of the left face, upper extremity, and lower extremity. His NIH stroke score was 16. MRI and MRA studies revealed occlusion of the right middle cerebral artery with restricted diffusion in the right basal ganglia and the posterior lateral aspect of the caudate head (Figure 1). There was mass effect on the right lateral ventricle and midline shift. Our information was that 6 h had elapsed since the onset of neurologic symptoms. Taking into account the possibility of a devastating clinical outcome and in conjunction with adult stroke and interventional radiologist specialist, the decision was made to attempt to restore arterial patency. 

**Figure 2:**
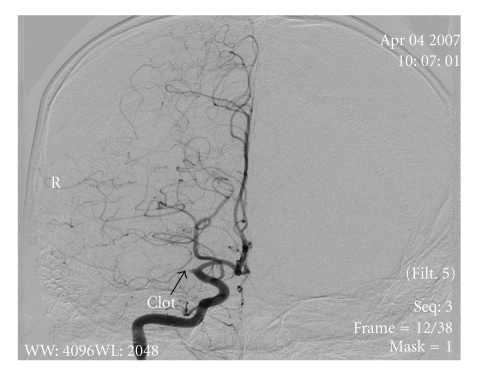
Coronal view of angiography arrow pointing to the area the Middle Cerebral Artery occlusion.

**Figure 3:**
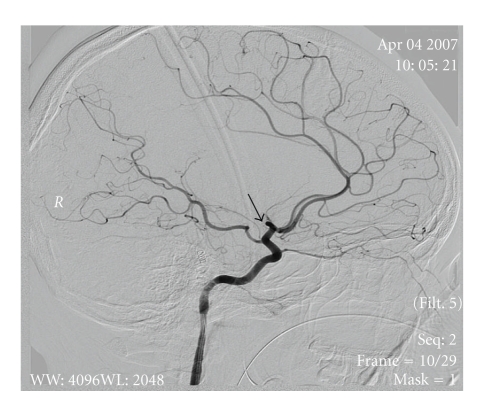
Sagittal view of angiography arrow pointing to the area the Middle Cerebral Artery occlusion.

**Figure 4:**
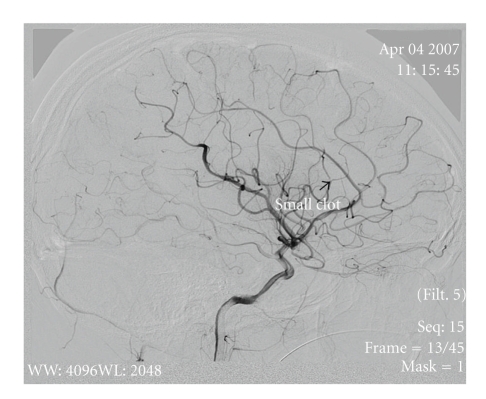
Sagittal view demonstrating a significant improvement in blood flow after rt-PA Abciximab and nitroglycerin were infused. Arrow points to a small residual occlusion.

**Figure 5:**
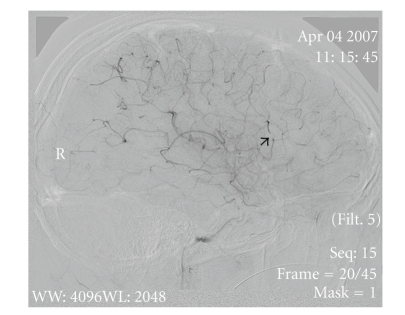
Sagittal view demonstrating a significant improvement in blood flow after rt-PA Abciximab and nitroglycerin were infused. Arrow points to a small residual occlusion.

The patient was anesthetized, intubated, and cooled to 33–35°C (mild therapeutic hypothermia). A thrombus was located (Figures 2 and 3); endovascular mechanical embolectomy with a Merci retrieval device was attempted without success. Recombinant tissue plasminogen activator (rt-PA) was infused into the MCA in doses of 0.7, 2, and 4 mg with minimal improvement in blood flow. Abciximab and nitroglycerin (25 *μ*g) were then infused with considerable improvement in blood flow (Figures 4 and 5). 

The patient was transferred to the pediatric ICU and remained sedated and mechanically ventilated. Neurosurgical consultation was obtained for an intracranial pressure (ICP) monitoring, but this was not placed due to recent thrombolytic administration and the initiation of systemic anticoagulation. Heparin was administered for 7 days, Abciximab (10 *μ*g/min for the first 12 h), aspirin (325 mg/day), magnesium sulfate (2000 mg/24 hrs), 25% albumin (1 g/kg IV every 6 h for the first 5 days), and memantine HCL (10 mg nasogastric each day for 3 days). Mild therapeutic hypothermia was maintained for 4 days to decrease acute postischemic cerebral edema followed by passive rewarming over a 24-hour period. A CT scan obtained at 24 hrs showed a decrease in the midline shift.

The central venous pressure (CVP) was maintained at 8–10 cm H_2_O and mean arterial pressure (MAP) at 75–80 mm Hg. A dual channel near infrared spectroscopy (NIRS) probe was applied to the scalp overlying both frontal lobes. Initially, there was a discrepancy between the two sides with the ischemic right side 10%–15% lower than the left side (40%–50% versus 65%–70%). Increasing CVP or MAP served only to augment the discrepancy. Subsequently, the partial pressure of arterial carbon dioxide was allowed to increase from 35–40 to 46–55 mm Hg with resolution of the discrepancy (both sides equal to 60%–65%). 

MR studies on the 7th day revealed appropriate MCA blood flow and partial resolution of the ischemic changes in the basal ganglia. Mass effect and right lateral ventricle compression by the basal ganglia were improved. He was extubated on day 10 and was able to communicate verbally, written, and receptive but had a small degree of residual left hemiparesis. He was transferred to a rehabilitation facility on day 15 where he exhibited an almost complete recovery within the following two weeks and was discharged home. Subsequent followup by our pediatric neurology service over the next year has demonstrated complete recovery to baseline without deficits. One year later he is taking advanced placement classes in school, playing junior high school basketball and baseball, and having no demonstrable neurological deficits.

**Figure 6:**
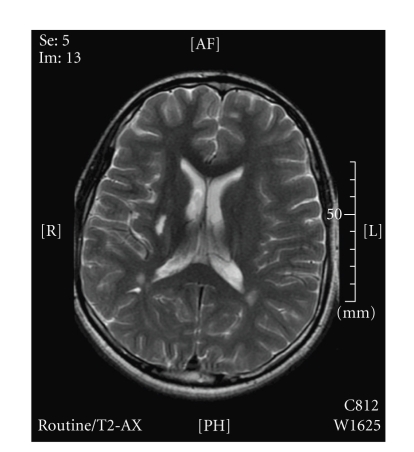
MRI 3 month after the initial insult showing a residual hyperdense signal abnormally in the periventricular region.

Cerebral angiography and serologic studies did not find the presence of dissection, vasculitis, or autoimmune disease; transthoracic echocardiography ruled out structural heart disease, intracardiac thrombus, and right to left intracardiac shunt, and hemoglobin electrophoresis, hypercoagulability studies, and lipid panels were all unremarkable. Doppler studies of the veins of the lower extremities and pelvis were all negative for deep venous thrombosis. An etiology for this patient AIS has not been found. An MRI performed 3 months after the events showed resolution of the edema and restricted diffusion with a residual hyperdense signal abnormally in the periventricular region (Figure 6).

## 3. Discussion

Our case report differs from others in the literature in that intra-arterial thrombolysis was undertaken 6 h after the onset of symptoms than most other reports of anterior circulation occlusion and few other patients have made as complete a recovery. Several provocative issues relevant to AIS in children are raised by his management: (a) the use of hyperacute therapies, (b) the role of antiplatelet agents and anticoagulants in the management of AIS, and (c) the role of adjunctive pharmacological neuroprotective agents. 

The growing recognition that AIS in adults is reversible if treated in a prompt manner has fostered a sense of urgency in clinicians who regard this condition as a “brain attack” and hospitals preparedness with designation of stroke centers for rapid triage and treatment. The resolve of the physician has a decisive effect on the timely implementation of therapies for stroke in adults [3].

By comparison, little sense of urgency to aggressively restore vascular patency exists for pediatric patients, and with the exception of AIS in the setting of sickle cell disease and Moyamoya disease, therapeutic approaches that embrace supportive care and rehabilitation are more common in pediatric hospitals. Development of a similar sense of determination and urgency is hampered by several obstacles. First, pediatric emergency room practitioners encounter AIS infrequently, and it is therefore not uncommon for considerable delay to occur in the diagnosis excluding the patient from the accepted time windows for hyperacute therapies. Second, the outcome of children with stroke is incorrectly thought to be better than that of adults. The outcome of AIS is mainly determined by size, location, and etiology of stroke. While the pediatric mortality is described to be between 5%–10% and is favorable when compared to adult patients, pediatric survivors of AIS are left with persistent neurologic and psychosocial deficit or seizure disorder for the rest of their lives [7, 8]. Third, the pathophysiology underlying the heterogeneous disorders of pediatric AIS makes it difficult to extrapolate the adult AIS literature to children, and many children's hospitals do not have preplanned algorithms for rapid diagnosis and treatment of AIS. Finally, many practitioners are uncomfortable administering thrombolytic medications in the absence of pediatric-specific data or clinical guidelines to support their use. Indeed, most recommendations for the treatment of AIS in children are derived from consensus and expert opinion, and neither the Royal College of Physicians (RCP) in the United Kingdom nor the American College of Chest Physicians (ACCP) in the United States recommends the administration of rt-PA to children with AIS [9, 10]. Despite this reluctance, several case reports to date have described their use in children [11–16], and a review of a national inpatient database registry indicates that a small minority of pediatric patients with AIS are receiving thrombolytic therapy [17, 18]. 

### 3.1. Thrombolysis/Embolectomy

The term “hyperacute therapies” has been coined to refer to therapies which must be initiated within an acceptable time interval in order to be effective, and they are designed to restore blood flow through obstructed vascular beds. Hyperacute therapies that are offered to adults include intravenous thrombolysis with rt-PA, intra-arterial thrombolysis with rt-PA, and mechanical embolectomy. 

The use of intravenous thrombolysis is now well established in the literature and is recommended for the treatment of AIS in adults without contraindications who present to medical attention within 3 h of symptoms onset [4, 5]. Patients treated with 0.9 mg/kg rt-PA (10% as a bolus and the remainder infused over 1 hour) had better functional outcomes at 3 months when compared to patients who received placebo, and mortality rates were similar between the two groups. Symptomatic intracranial hemorrhage occurred in 6.4% of patients treated with rt-PA and 0.6% of controls. The time period from onset of stroke symptoms to beneficial treatment with thrombolytic medications remains a subject of continuous debate. The debate addresses the observation that as time from symptom onset increases the likelihood of outcome benefit from thrombolysis decreases while thrombolytic therapy is associated with a substantial increase in likelihood of intracranial hemorrhage. However, a recent publication demonstrated benefit while extending the administration up to 4.5 h after the onset of symptoms [6]. Furthermore, whether strict adherence to pre-defined time interval cutoffs should be used to determine whether thrombolytic therapy should be offered is unclear. Comparison of MRI diffusion-weighted imaging (DWI) and perfusion-weighted imaging (PWI) may allow the clinician to determine for each patient how much brain tissue is receiving inadequate blood flow (abnormal PWI) to still viable brain tissue (intact DWI). Such an examination might allow clinicians to determine for patients presenting after more prolonged time intervals from onset of neurologic symptoms whether thrombolytic therapy could still be beneficial to a given patient [10, 11, 18, 19]. 

Intra-arterial administration of thrombolytics (urokinase and rt-PA) is sometimes offered as a potential, viable alternative for the delivery of thrombolytic medications to obstructed cerebral vascular beds for patients who are not candidates for systemic intravenous thrombolysis or those who present to medical attention between 3 and 6 h after symptom onset. Intra-arterial administration of recombinant prourokinase and intravenous heparin within 6 h of onset of stroke symptoms resulted in improved functional outcomes when compared to intravenous heparin alone [20], but intra-arterial and intravenous thrombolyses have not been compared to each other. 

Other options for recanalization of obstructed cerebral vasculature include the use of endovascular mechanical retrieval devices for clot removal. Intra-arterial and endovascular mechanical embolectomies have extended the time window for treatment up to 8 h for AIS in the anterior circulation, and even longer in cases involving the posterior circulation. While mechanical intra-arterial thrombectomy has demonstrated a high recanalization rate [21], randomized trials that describe functional outcome are lacking. 

There is evidence that thrombolytic medications are being administered to 1.6% of the children with AIS, and this data suggests that children may have inferior clinical outcomes with respect to mortality [18]. However, it cannot be determined from this data whether inferior outcomes are related to AIS severity or the treatment itself. Pediatrics encompasses a wide range of ages with maturational differences. Optimal rt-PA dosing is likely to differ as there are age-dependent differences in hemostasis [22, 23], younger individuals demonstrating diminished specific indices of fibrinolysis and global, increased fibrinolytic capacity [22]. Case reports over the past eight years have described the administration of thrombolytic medications to pediatric patients with good result at widely different interval from onset of symptoms [13, 16, 24–29]. Location of the AIS and anticipated neurological deficits is a major consideration. Indeed, due to the dire outcome of vertebrobasilar thrombosis (VBT), case reports in the pediatric literature describe successful administration of thrombolytic therapy 17 h after the onset of symptoms [24, 25].

#### 3.1.1. Anticoagulation and Antiplatelet Therapy

The role of anticoagulation in pediatric AIS patients is unclear. The RCP and ACCP guidelines provide recommendations for acute treatment and secondary prevention of AIS. Their recommendations differ with respect to the role of anticoagulation in the acute management of AIS in children. RCP guidelines for the management of acute AIS in children (not associated with intracranial hemorrhage or sickle cell disease) recommend the administration of aspirin (5 mg/kg/day) [10]. The ACCP guidelines for management of acute AIS in children have a Class II recommendation in regard to the initiation of unfractionated heparin or low-molecular weight heparin (LMWH) for 5–7 days or until cardioembolic stroke and vascular dissection have been excluded [2, 9, 10]. Heparin may be safe for the treatment of strokes secondary to sinovenous thrombosis even though studies in regard to efficacy are lacking. The ACCP points out that while LMWH may offer reproducible pharmacokinetics and fewer monitoring tests in comparison with unfractionated heparin the effects of LMWH cannot be rapidly reversed after its discontinuation [2]. Adult guidelines from the AHA recommend the administration of aspirin (325 mg/day) within 24–48 h of stroke onset [4, 5]. 

For secondary prevention of AIS recurrence the ACCP recommends initiation of aspirin therapy (2–5 mg/kg/day) after discontinuation of anticoagulation [9]. For children with vascular dissection or cardioembolic stroke the ACCP recommends continuation of anticoagulation with low-molecular weight heparin or vitamin K antagonists for 3–6 months [9]. The RCP recommends continuation of aspirin (1–3 mg/kg/day) for all children except those with Moyamoya disease, sickle cell disease, or arterial dissection [6]. The RCP recommends treatment with anticoagulation for prevention of secondary AIS in patients with arterial dissection or cardioembolic stroke [10].

While aspirin is the major antiplatelet agent in clinical use, the roles for other antiplatelet and anticoagulant medications are under investigation. Adjuvant medications such as thrombin inhibitors and inhibitors of platelet glycoprotein IIb/IIIa (GIIb/IIIa) are being evaluated as monotherapy and in combination with rt-PA for the treatment of acute ischemic stroke. Current studies also evaluate the use of argatroban (a thrombin inhibitor) [30], ancrod (a viper-derived enzyme that cleaves fibrinogen and promotes endogenous release of plasminogen activator) [31, 32], and desmoteplase (a bat saliva-derived protease similar in function to human plasminogen activator) [33, 34]. A recently completed phase III trial with an intravenous administration of a glycoprotein IIb/IIIa antagonists, Abciximab, did not demonstrate safety or efficacy with an increased incidence of intracranial hemorrhage [35].

#### 3.1.2. Attempts to Prevent Secondary Neuronal Injury

The literature is not clear how to prevent secondary injury in AIS. Once blood flow through the MCA was restored, and measures were taken to protect the ongoing patency of the vessel, we next considered measures that attempted to minimize secondary neuronal injury during reperfusion of the ischemic regions of the brain. Included among these were (a) the maintenance of adequate cerebral blood flow, (b) the prevention of hyperglycemia, (c) the continuation of mild therapeutic hypothermia, and (d) administration of 25% albumin, magnesium salts, and N-methyl-D-aspartate receptor (NMDA) antagonists.

Maintenance of adequate cerebral perfusion pressure may be of importance in optimizing neurologic outcome after AIS if regional autoregulation is lost or in cases of severe increase in intracranial pressure. While verification of adequacy of cerebral perfusion pressure might have been optimized with the assistance of an ICP monitor, the risks of ICP monitor placement in this anticoagulated child were felt to be prohibitive. In the absence of ICP monitoring we assumed that our patient probably had at least moderately increased ICP. Accordingly, we allowed moderate permissive hypertension, maintained a high osmolarity with hypertonic saline and mild hypothermia. Fever should be aggressively treated [36] in AIS, and mild hypothermia has shown to decreases acute postischemic cerebral edema, a transient phenomenon that is maximal at 2–4 days postinfarct [37–39]. However it has been difficult to ascertain a positive impact in outcome with its use, and a large randomized study is needed [40]. A rebound in intracranial pressure can occur when rewarming a patient that was hypothermic.

As an indirect monitor of the adequacy of cerebral perfusion, near infrared spectroscopy (NIRS) monitoring was applied to both cerebral hemispheres. In the initial h, regional cerebral oxygen saturation (rSO_2_) was significantly lower in the compromised hemisphere. The stepwise preload augmentation and addition of norepinephrine to augment mean arterial pressure only increased the differences as a “steal” phenomenon by the nonaffected area. The subsequent introduction of mild permissive hypercapnia produced an increase and equalization of rSO_2_ in both hemispheres.

While these trends in rSO_2_ could be interpreted to indicate the restoration of adequate oxygen supply/demand in the affected hemisphere, prior studies employing NIRS in the noninvasive monitoring of patients with middle cerebral artery infarction and cerebral edema suggest that disappearance of the rSO_2_ difference between hemispheres may indicate worsening cerebral edema and herniation in the infarcted hemisphere [41]. Computed tomography of the brain on day 3 and reassuring neurologic examinations during improving rSO_2_ values in the affected hemisphere assured us that the disappearance of the rSO_2_ gradient between hemispheres was not due to an ominous process. The role of NIRS as a noninvasive monitor of cerebral circulation in pediatric stroke remains to be delineated.

#### 3.1.3. Other Potentially Neuroprotective Pharmacologic Interventions

Our patient was treated with scheduled infusions of hypertonic albumin after returning from the angiography suite. The administration of hypertonic solutions of albumin is recommended for the treatment of adult ischemic stroke patients in our institution and is based on studies that demonstrate improved functional neurologic outcome in adult patients suffering from ischemic stroke [42, 43]. Preclinical and phase I and II trials of the administration of albumin in acute ischemic stroke suggest that the time to administration of albumin after ischemic stroke is critically important (within 4-5 h) [44] and that a synergistic effect may exist when rt-PA is also administered [42]. The physiologic benefit of albumin administration is believed to be related to its effect on the endothelium [45–49], blood viscosity and aggregation [50], interactions with nitric oxide [51], and inhibition of platelet aggregation [52, 53]. Studies of albumin administration in experimental models of central nervous system ischemia demonstrate that albumin administration rapidly improves blood flow in cerebral vessels with critically reduced flow and clears thrombotic material adherent to the endothelium in ischemic venules [53, 54]. While preliminary studies of high dose albumin administration to AIS patients have been encouraging, the results of the multicenter, randomized, placebo-controlled efficacy trial of albumin in acute ischemic stroke—ALIAS Phase III trial is underway and may provide definitive answers on the role of albumin therapy in adult AIS. Pediatric trials on the use of albumin in this setting are still lacking. Hypertonic albumin administration may induce pulmonary edema in adults; however it is frequently utilized in pediatric intensive care in patients with other conditions without deleterious effects. Decompressive craniotomy is an effective treatment to treat increased intracranial pressures in patients with extensive stroke [55]. 

We administered memantine HCl and magnesium sulfate on a scheduled basis. This is the routine practice of our adult stroke team, and their administration is predicated on several theoretical principles. The magnesium ion regulates cellular energy metabolism, vascular tone, and cell membrane ion transport [56]. Magnesium may regulate ATP concentrations and is a prerequisite for ATP regeneration after ischemia and reperfusion [57, 58]. The magnesium ion also blocks the ion channel of the N-methyl-D-aspartate (NMDA) receptor in a voltage-dependent fashion, and increasing extracellular magnesium concentrations in vitro cause noncompetitive NMDA blockade [59, 60]. In vitro and in vivo models of focal and global ischemia have demonstrated neuronal protection that in some instances is as great as that seen with noncompetitive NMDA antagonists. Magnesium protects both hippocampal neurons from glutamate-mediated necrosis and white matter tracts from prolonged ischemia [61]. Systemic MgCl_2_ decreased cerebral infarct volume by 20% after permanent middle cerebral artery occlusion in the rat [62]. Memantine hydrochloride, another NMDA receptor antagonist, was also administered to attenuate reperfusion injury. While a neuroprotective role has been ascribed to magnesium sulfate and NMDA receptor antagonists, no data from human trials have shown conclusive benefit to adult patients with AIS, and data is therefore certainly lacking to make recommendations regarding use in pediatric AIS.

## 4. Conclusions

The case described is a paradigmatic observation of the possibility of using hyperacute therapies in children with strokes: early recognition of the event, probable thromboembolism mechanism, MCA distribution, and rapid consultation with multiple specialists were crucial factors influencing the decision process. Thrombolysis is probably less favorable when other types of vasculopathy, that most often affect young children (e.g., basal arterial stenosis), are present. There is a need to increase awareness of childhood stroke by pediatricians and emergency room physicians caring for children with the aim of reducing the time lag from symptoms to the correct diagnosis. It is imperative to have in place a preplanned diagnostic algorithm as well as therapeutic algorithm. Most of emergency departments do not receive regularly children with stroke. Except in very specific cases, it is thus not possible to build autonomic pediatric stroke centers. Nevertheless, as it is well documented in the present case report, collaboration between emergency room physicians, pediatric neurologists, and physicians working in adult stroke centers should be highly considered.
